# Impact of geriatric covariates on metronomic cyclophosphamide efficacy and tolerance—the CHEMETROLD study

**DOI:** 10.1093/oncolo/oyag130

**Published:** 2026-04-13

**Authors:** Clara Griffe, Christine Ravot, David Dayde, Claire Falandry

**Affiliations:** Institut du Vieillissement, Hospices Civils de Lyon, Lyon 69002, France; Service de Gériatrie, Centre Hospitalier Lyon Sud, Hospices Civils de Lyon, Pierre-Bénite 69495, France; Plateforme de recherche clinique de l‘Institut de Cancérologie des Hospices Civils de Lyon, Centre Hospitalier Lyon Sud, Hospices Civils de Lyon, Pierre-Bénite 69495, France; Université Lyon1, INSERM, INRAE, CarMeN, Pierre-Bénite 69495, France; Service de Gériatrie, Centre Hospitalier Croix-Rousse, Hospices Civils de Lyon, Lyon 69004, France

**Keywords:** metronomic chemotherapy, cyclophosphamide, advanced cancer, geriatric oncology, frailty

## Abstract

**Background:**

Metronomic cyclophosphamide is sometimes considered in older or frail patients with advanced cancer when disease-related symptoms affect quality of life and standard treatments are no longer suitable. While clinical feedback on its tolerance and efficacy is often positive, the evidence remains limited in geriatric, comorbid populations.

**Patients and methods:**

This retrospective, monocentric study evaluated the impact of patient-, tumour-, and treatment-related characteristics on the efficacy and safety of metronomic cyclophosphamide in patients aged ≥70 years with metastatic solid tumors. Baseline geriatric assessments were collected, and treatment exposure, tolerance, and outcomes were analyzed. An exploratory subgroup analysis focused on patients treated for ≥9 months.

**Results:**

Thirty-seven patients (median age 84 years, range 70-96) were included: 15 had prostate cancer (40.5%), 8 breast cancer (21.6%), and 14 other primary tumors (37.8%). The median treatment duration was 4 months; the median progression-free survival was 4 months and 6 months in breast and prostate cancer subgroups. Overall dose intensity was 87%, with 62.2% of patients maintaining ≥90%. Treatment was generally well tolerated; only 5 grade ≥3 toxicities were reported, and 83.7% of patients remained at home during therapy. Notably, patients treated for ≥9 months had a poorer performance status and lower functional scores at baseline. No specific molecular alterations were associated with prolonged treatment benefit.

**Conclusion:**

Metronomic cyclophosphamide appears to be a safe and potentially effective option in older and frail patients with metastatic cancer, including those with limited functional reserve. These findings support its use as a pragmatic alternative to supportive care, even in late disease stages.

Implications for practice

- Metronomic cyclophosphamide was well tolerated in a frail and highly comorbid population, with high treatment adherence and dose intensity, suggesting its feasibility even in vulnerable patients.
- Disease control rates were comparable to those observed in younger cohorts from previously published studies.
- Patients who remained on treatment for ≥9 months presented with poorer functional status at baseline, suggesting that functional impairment may not preclude potential benefit.
- Metronomic cyclophosphamide may offer a valuable therapeutic option beyond supportive care in older patients with advanced solid tumors who are unfit for standard chemotherapy.

## Introduction

As life expectancy increases, so does the incidence of age-related diseases, including cancer. After age 80, cancer risk rises significantly,^1^ partly due to age-associated immune alterations such as chronic low-grade inflammation^2^ (“inflammaging”) and impaired adaptive immunity.^3^ These changes, combined with multimorbidity^4^ and polypharmacy, make cancer management particularly challenging in older adults. Metronomic chemotherapy, defined as the regular administration of low-dose cytotoxic agents without prolonged breaks,^4^ has shown immunomodulatory^5^ and antiangiogenic effects^6^ distinct from traditional cytotoxic regimens. Cyclophosphamide is the most commonly used agent in this setting, with reports of favorable tolerance^7–9^ and modest efficacy in breast, prostate, and gynecologic cancers^10^ using starting doses ranging from 25 to 100 mg per day.^11^ However, evidence in geriatric and highly comorbid populations remains limited. In clinical practice, metronomic cyclophosphamide is often considered in late treatment lines, especially when standard regimens are no longer appropriate due to poor performance status or frailty. Despite encouraging anecdotal outcomes, data are lacking in supporting its systematic use in this population. This retrospective study aimed to evaluate the safety and efficacy of metronomic cyclophosphamide in a cohort of older patients with metastatic solid tumors, and to identify clinical and geriatric factors potentially associated with treatment benefit.

## Materials and methods

### Study design and population

This retrospective monocentric study included patients aged ≥70 years treated with metronomic oral cyclophosphamide for metastatic solid tumors at Lyon Sud Hospital between November 2011 and March 2022. Ethical approval was obtained (HCL authorization n°22-5085).

### Data collection

Baseline data included age, sex, cancer type, treatment line, performance status (PS), and cyclophosphamide starting dose. Geriatric covariates were extracted from initial assessments: CIRS-G score,^12^ BMI, MNA,^13^ ADL, IADL,^14^ MMSE,^15^ and living status. Comorbidities were categorized (eg, cardiac, renal, pulmonary). Follow-up data included treatment modifications, lab parameters (albumin, hemoglobin, leukocytes, platelets), and reasons for discontinuation.

### Outcomes

The primary objective was to assess treatment efficacy and safety. Secondary analyses explored associations between clinical, geriatric, and treatment factors and treatment duration. A subgroup of patients receiving treatment ≥9 months was analyzed in detail. When available, tumor samples from these patients were assessed by next-generation sequencing (NGS).

### Statistical analysis

Progression-free survival (PFS) and overall survival (OS) were analyzed using the Kaplan-Meier method, with median values and corresponding 95% confidence intervals (CIs) reported. Comparisons between long and short treatment durations used Fisher’s exact and Mann-Whitney tests, as appropriate.

### Safety evaluation

Due to the retrospective nature of the study, safety was assessed based on total treatment exposure, reasons for discontinuation, number and duration of treatment suspensions, and dose intensity. Reasons for treatment interruptions were classified into 4 categories: acute events (infectious complications or comorbidity decompensation not directly related to cancer); general adverse events (systemic symptoms such as fatigue, anorexia, weight loss, and malnutrition); hematological events (anemia, thrombocytopenia, and leukopenia); miscellaneous (other toxicities, including hepatic and gastrointestinal side effects). Malnutrition was defined as BMI <22 kg/m^2^; severe malnutrition was defined as BMI <20 kg/m^2^ or serum albumin <30 g/L. Hematologic toxicities were graded according to CTCAE v5.0. Nursing home admission during follow-up was recorded as a marker of functional decline. Treatment discontinuation was categorized as due to tumor progression, general adverse events, acute events, hematologic toxicity, death, or long-term disease control. Dose intensity was calculated based on a standard dose of 50 mg/day, expressed as a percentage of planned dose delivered over time.

## Results

### Patient characteristics

A total of 37 patients were included: 17 men (46%) and 20 women (54%), with a median age of 84 years (range 70-96). Most patients had a performance status (PS) of 1 or 2 (73%), while 2 patients (5.4%) had a PS of 3. Tumor types were as follows: prostate cancer (*n* = 15, 40.5%), breast cancer (*n *= 8, 21.6%), and other solid tumors (*n *= 14, 37.8%) including ovarian (*n *= 5), endometrial (*n *= 5), bladder (*n *= 2), gastric (*n *= 1), and cancer of unknown primary (*n *= 1). Most patients had received at least 3 prior lines of treatment. Geriatric assessment at baseline showed a CIRS-G score ≥10 in 24 patients (64.9%). Nine patients (24.3%) had a BMI ≤22 kg/m^2^, and 4 were already living in nursing homes. Half of the cohort had an ADL score ≤5/6 and an IADL score ≤5/8 (see Table 1).

**Table 1:** Patients’ characteristics of the CHEMETROLD study population.

Characteristics	Number of patients (%)	Characteristics	Number of patients (%)
**Gender**		Comorbidities	
**Male**	17 (46.0)	Diabetes	8 (21.6)
**Female**	20 (54.0)	Hypertension	22 (59.5)
**Age (years)**		Heart disease	14 (37.8)
**Median**	84	Pulmonary disease	4 (10.8)
**Range**		Chronic kidney disease	6 (16.2)
**WHO performance status**		CIRS-G	
**0**	1 (2.7)	Mean score	10.7
**1**	17 (46.0)	Level 3 or 4 comorbidities:	
**2**	10 (27.0)	1	23 (62.1)
**3**	2 (5.4)	2	13 (35.1)
**Unknown**	7 (18.9)	3	1 (2.7)
**Cancer setting**		MMSE score	
**Prostate**	15 (40.5)	Mean	25.06
**Breast**	8 (21.6)	≤24/30	25 (67.6%)
**Ovaries**	5 (13.5)	ADL score	
**Uterus**	5 (13.5)	Mean	4.66
**Other**	4 (10.8)	0	1 (2.7)
**Treatment line**		1	2 (5.4)
**1**	3 (8.1)	2	4 (10.8)
**2**	9 (24.3)	3	3 (8.1)
**3**	15 (40.5)	4	1 (2.7)
**4**	4 (10.8)	5	6 (16.2)
**≥ 5**	6 (16.22)	6	20 (54.05)
**BMI (kg/m²)**		IADL score	
**Mean**	25.1	Mean	4.62
**≥ 22**	28 (75.7)	0	2 (5.4)
**< 22**	9 (24.3)	1	8 (21.6)
**MNA**		2	2 (5.4)
**≥ 12**	3 (8.1)	3	1 (2.7)
**<12 and ≥ 8^a^**	20 (54.1)	4	5 (13.5)
**< 8^b^**	12 (32.4)	5	2 (5.4)
**Residence**		6	2 (5.4)
**Private home**	33 (89.2)	7	6 (16.2)
**Nursing home**	4 (10.8)	8	9 (24.3)

a At risk of malnutrition.

b Malnourished.

Abbreviations: ADL: activities of daily living; BMI: body mass index; IADL: instrumental ADL; MMSE: mini mental status evaluation; MNA: Mini Nutritional Assessment; WHO: World Health Organisation.

### Efficacy

**Figure 1:**
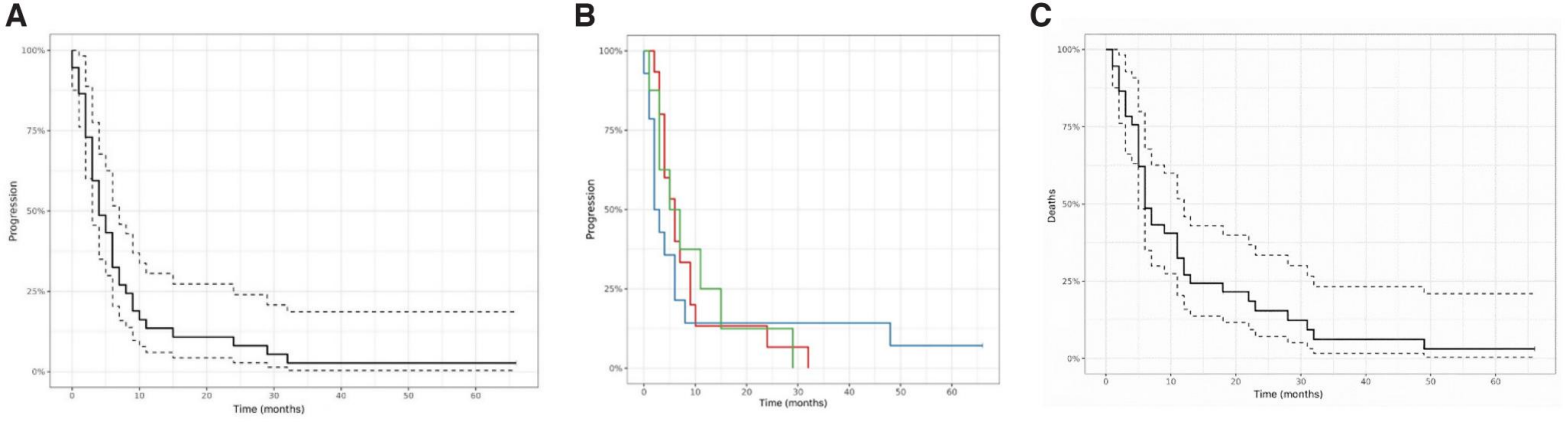
Kaplan Meier estimates of survival in CHEMETROLD study: progression-free survival in the whole population (A), according to the cancer type (B, green: breast cancer; red: prostate cancer; blue: other primaries) and overall survival (C).

The median treatment duration was 4 months (mean 6.4 months), ranging from less than 1 month to 29 months. The median time to progression was 4 months for the overall population. Subgroup analysis showed a median progression-free survival (PFS) of 6 months in breast and prostate cancer patients, and 2.5 months in other tumor types; however, these differences were not statistically significant. Median overall survival (OS) was 6 months (Figure 1).

### Tolerance and safety

**Figure 2:**
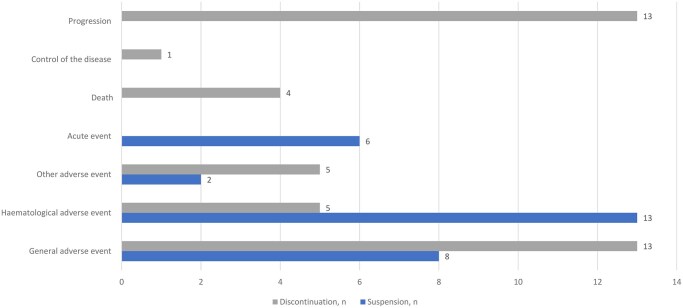
Reasons for suspension or discontinuation of treatment within CHEMETROLD study.

Treatment suspensions occurred in 13 patients, with 7 experiencing multiple interruptions. The most common causes of suspension were hematological (44.8%) and general adverse events (27.6%). Six suspensions were due to acute events. A total of 41 treatment discontinuations were recorded, for cancer progression (*n *= 13, 31.7%); general adverse events (*n *= 13, 31.7%); acute adverse events (*n *= 5, 12.2%); hematological toxicity (*n *= 5, 12.2%); death during treatment (*n *= 4, 9.8%); discontinuation despite disease control (*n *= 1). Acute adverse events included 2 hepatic toxicities, 2 gastrointestinal intolerances, and 1 acute renal failure (Figure 2). In total, 51 toxicities were reported: 27 were grade 1 (52.9%) and 5 were grade ≥3 (9.8%). Hematologic supportive care (erythropoietin or transfusion) was required for 11 patients (29.7%). Malnutrition was obseved in 9 patients (24.3%) at baseline (all meeting criteria for severe malnutrition), in 14 patients (37.8%) at first follow-up, and in 16 patients (43.2%) at the end of treatment. Among those, 13 patients (35.1%) had severe malnutrition. Despite this, 31 patients (83.7%) remained at home throughout the treatment. Only 2 patients required admission to a nursing home by the end of treatment, both due to cancer progression.

### Treatment exposure

**Figure 3:**
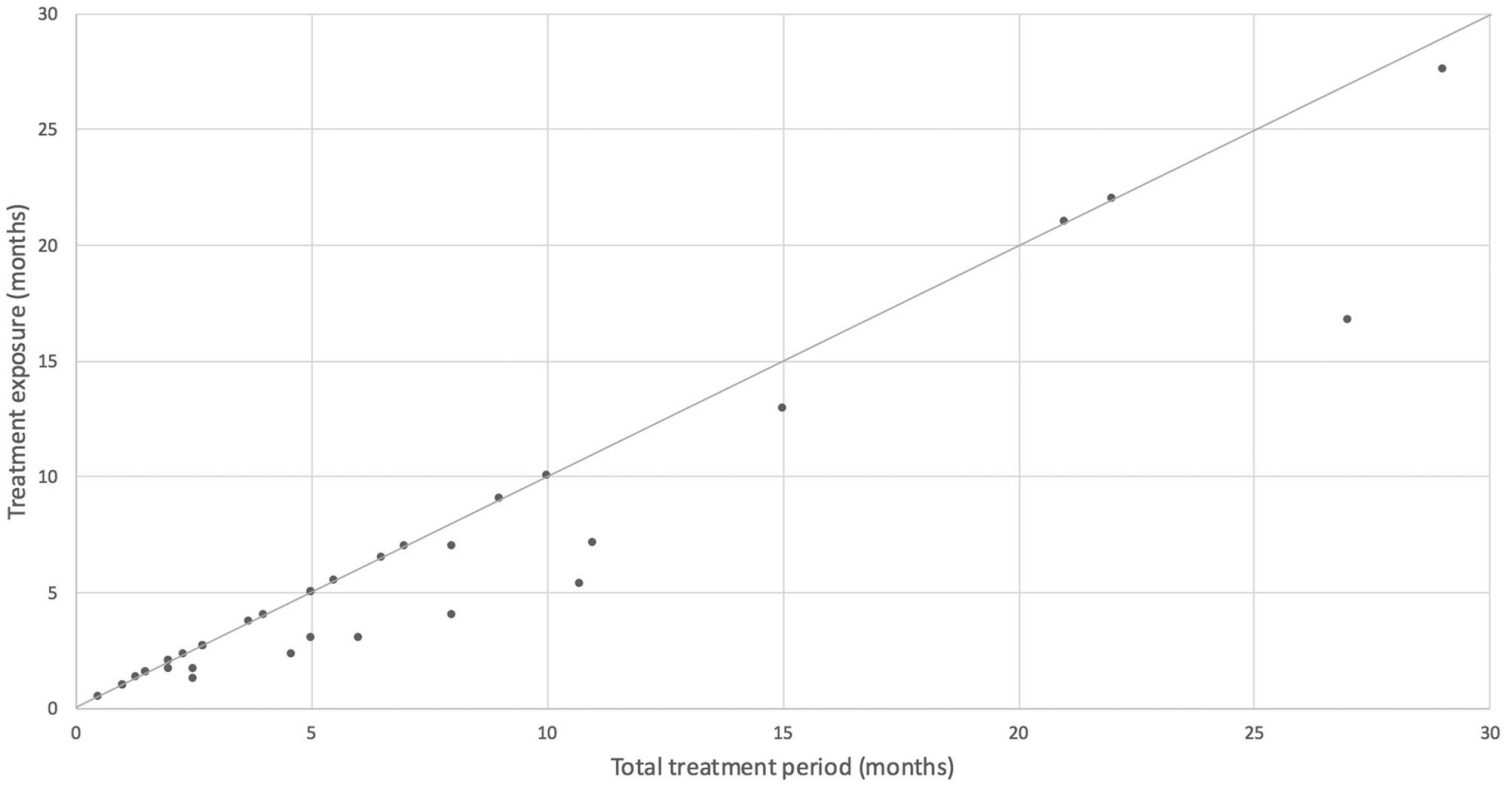
Treatment exposure relative to the total treatment period in CHEMETROLD study.

Most patients started with 50 mg/day of cyclophosphamide. Two patients began at alternating doses of 50 mg and 100 mg/day. Four patients required dose reductions due to toxicity: 1 patient received 50 mg/day for 3 weeks out of 4 (asthenia); 3 patients received 50 mg every other day due to hematologic toxicity (*n *= 2) or renal failure (*n *= 1). The overall dose intensity was 87%; 23 patients (62.2%) maintained a cumulative dose intensity ≥90%, while 6 patients (16.2%) had an intensity ≤50%. Patients with prolonged disease control did not experience longer cumulative treatment interruptions compared to others (Figure 3).

### Patients with prolonged treatment duration

Among the 8 patients (21.6%) treated for at least 9 months, 6 (75%) had received ≥ 3 treatment lines, 5 (55.6%) had a CIRS-G score ≥10, and 2 (22.2%) had a *PS* ≥3. Compared with the rest of the cohort, these patients tended to be older, had significantly lower IADL scores, and a more impaired performance status at baseline (see Table 2). However, primary tumor types were similar between groups.

**Table 2:** Oncologic and geriatric characteristics at treatment initiation of patients with long (≥ 9 months) versus shorter response to metronomic cyclophosphamide in CHEMETROLD study.

		Short responders (*n* = 29)	Long responders (*n* = 8)	*n*	*p*	test
**Cancer type, *n***		11 (38%)	4 (50%)	15	0.28	Fisher
**Prostate**		5 (17%)	3 (38%)	8		
**Endometrial**		5 (17%)	0 (0%)	5		
**Ovarian**		5 (17%)	0 (0%)	5		
**Bladder**		2 (6.9%)	0 (0%)	2		
**Stomach**		1 (3.4%)	0 (0%)	1		
**Unknown**		0 (0%)	1 (12%)	1		
**Age** **median [Q25-75]**		83.0 [78.0; 87.0]	87.0 [86.3; 88.0]	37	0.09	Mann-Whitney
**PS** **median [Q25-75]**		1.00 [1.00; 1.50]	2.00 [2.00; 2.50]	30	**0.003**	Mann-Whitney
**Initial albumin (g/L)** **median [Q25-75]**		34.7 [28.9; 38.5]	29.1 [22.3; 36.6]	24	0.48	Mann-Whitney
**CIRS-G total score** **median [Q25-75]**		10.0 [9.00; 13.0]	10.5 [9.00; 11.3]	37	0.64	Mann-Whitney
**ADL score** **median [Q25-75]**		6.00 [5.00; 6.00]	4.00 [2.00; 6.00]	37	0.20	Mann-Whitney
**IADL score** **median [Q25-75]**		6.00 [3.00; 8.00]	1.00 [1.00; 4.80]	37	0.06	Mann-Whitney
**BMI** **median [Q25-75]**		24.2 [22.7; 27.2]	27.0 [18.5; 27.5]	37	0.42	Mann-Whitney
**MMSE** **median [Q25-75]**		26.0 [23.0; 28.0]	27.0 [19.2; 28.2]	35	0.75	Mann-Whitney
**MNA** **median [Q25-75]**		9.00 [6.00; 11.0]	7.00 [5.00; 9.80]	35	0.58	Mann-Whitney
**Gender, *n***	F	16 (55%)	4 (50%)	20	1.00	Fisher
	M	13 (45%)	4 (50%)	17		
**Heart disease, *n***	No	18 (62%)	6 (75%)	24	0.69	Fisher
	Yes	11 (38%)	2 (25%)	13		
**Diabetes, *n***	No	23 (79%)	6 (75%)	29	1.00	Fisher
	Yes	6 (21%)	2 (25%)	8		
**Hypertension, *n***	No	17 (59%)	6 (75%)	23	0.68	Fisher
	Yes	12 (41%)	2 (25%)	14		
**CKD, *n***	No	25 (86%)	6 (75%)	31	0.59	Fisher
	Yes	4 (14%)	2 (25%)	6		
**Pulmonary disease, *n***	No	27 (93%)	6 (75%)	33	0.20	Fisher
	Yes	2 (7%)	2 (25%)	4		
**Nutritional status, *n***	N	13 (52%)	5 (71%)	18	0.4	Fisher
	M	4 (16%)	1 (14%)	5		
	SM	8 (32%)	1 (14%)	9		

Abbreviations: ADL: activities of daily living; BMI: body mass index; IADL: instrumental ADL; M: malnutrition; MMSE: mini mental status evaluation; N: normal; PS: performance status; SM: severe malnutrition.

Tumor samples from 4 long-duration patients were available for next-generation sequencing (NGS) analysis. These included 2 infiltrating ductal breast carcinomas, 1 small-cell prostate carcinoma, and 1 oncocytic adenocarcinoma. Molecular alterations identified included: *BRCA2* mutation (homologous recombination pathway) in one of the breast cancers, *PTEN* mutation in the second, and *RB1*, *TP53*, and *ERBB2* mutations in the prostate cancer. These alterations are known to be involved in cell cycle regulation, DNA repair, and tumor proliferation. However, no consistent molecular profile was associated with longer response to metronomic cyclophosphamide.

## Discussion

This retrospective study provides a detailed analysis of patient-, tumor-, and treatment-related factors associated with the efficacy and safety of metronomic cyclophosphamide in an older, frail population with advanced cancer. In many such patients, best supportive care is often the only alternative, and continuing disease-directed treatment may raise concerns about medical overuse. This underscores the importance of evaluating the benefit-risk balance of metronomic chemotherapy in this specific setting. Despite the high burden of comorbidities and functional limitations at treatment initiation, metronomic cyclophosphamide was generally well tolerated. Adverse events were mainly mild to moderate in severity, with a relatively low incidence of grade ≥3 toxicities. Dose reductions and treatment suspensions were uncommon, and overall dose intensity remained high—suggesting good feasibility in this population. Furthermore, patients treated over longer periods did not experience more frequent or prolonged interruptions, indicating no evidence of cumulative toxicity. General adverse events—defined as fatigue, anorexia, weight loss, and malnutrition—were frequent, but their interpretation is complex. These symptoms may be driven by cancer progression, underlying frailty, or age-related physiological decline, rather than directly by the treatment itself. Mild anemia was the most common hematologic event, and often multifactorial in this population due to chronic kidney disease, nutritional deficiencies, or age-related marrow dysfunction. In terms of efficacy, disease control and progression-free survival were comparable to previously reported outcomes in younger, less comorbid cohorts treated with metronomic cyclophosphamide for breast or prostate cancer.^16^^,^^17^ Median progression-free survival was 4 months overall, and 6 months in breast and prostate subgroups. As expected, overall survival was shorter, likely reflecting the poorer general prognosis of this older and frail population.

**Figure 4:**
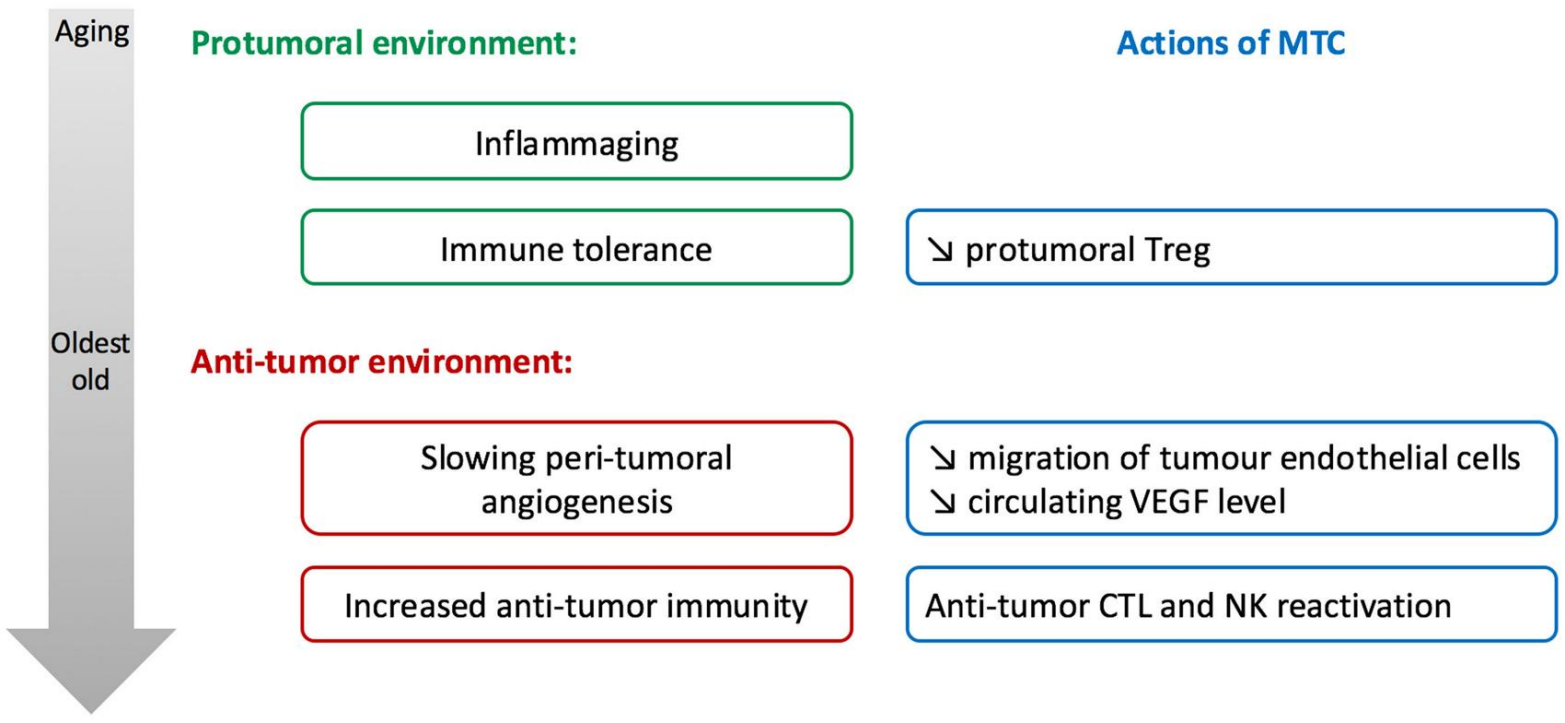
A proposed model to explain the influence of age on anti-tumoral properties of metronomic cyclophosphamide (MTC). Abbreviations: CTL: cytotoxic T lymphocytes.

To our knowledge, this is the first study focusing on metronomic cyclophosphamide in such a highly comorbid, geriatric oncology cohort. The small sample size and retrospective design represent limitations, as does the difficulty in objectively assessing disease progression in very frail patients, where imaging may not always be feasible or ethical. Some treatment discontinuations labeled as “tolerance-related” may in fact reflect undiagnosed cancer progression. An exploratory analysis of patients treated for ≥9 months revealed an unexpected association: these individuals had significantly poorer functional status and a trend toward older age and greater dependency at baseline. These characteristics are typically linked to poorer outcomes, which raises intriguing hypotheses. One possibility is that tumor progression is slower in the oldest and most frail individuals, due to age-related changes in the tumor microenvironment. Epidemiological studies have shown a decrease in cancer incidence after age 75,^18^ possibly due to reactivation of anti-tumor immunity,^19^ reduced angiogenesis,^20^ or slowed cellular metabolism. Metronomic chemotherapy is thought to exert part of its effect through immunomodulation and anti-angiogenic activity. In this context, it is plausible that metronomic cyclophosphamide may act synergistically with aging-related changes—such as chronic inflammation (inflammaging), altered immune cell composition, or reduced vascular remodeling—to inhibit tumor growth. This could explain the apparent paradox of prolonged disease control in patients with poorer baseline function. Next-generation sequencing of tumors from patients with extended treatment duration did not reveal a common molecular signature predictive of benefit. Identified alterations (eg, BRCA2, PTEN, TP53, RB1) are commonly found in advanced cancers and were not unique to this subgroup. This supports the hypothesis that microenvironmental or host factors, rather than tumor-intrinsic characteristics, may play a greater role in determining response in this setting (see Figure 4).

In conclusion, metronomic cyclophosphamide appears to be a well-tolerated and manageable treatment option for older and frail patients with advanced solid tumors, including those with poor functional status. Its efficacy in this population, often underrepresented in clinical trials, is encouraging and may be linked to the unique features of the aging tumor microenvironment and immune system. Our findings support the use of metronomic chemotherapy as a viable therapeutic strategy that balances efficacy and quality of life in a vulnerable patient group. Future prospective studies should further explore the immunological and biological mechanisms underlying treatment response in the elderly, aiming to optimize personalized care in this growing population.

## Data Availability

The data underlying this article cannot be shared publicly due to the privacy of individuals that participated in the study. The data will be shared on reasonable request to the corresponding author.
